# Routine Endoscopy for Esophageal Cancer Is Suggestive for Patients with Oral, Oropharyngeal and Hypopharyngeal Cancer

**DOI:** 10.1371/journal.pone.0072097

**Published:** 2013-08-15

**Authors:** Shih-Han Hung, Ming-Chieh Tsai, Tsai-Ching Liu, Herng-Ching Lin, Shiu-Dong Chung

**Affiliations:** 1 Department of Otolaryngology, Taipei Medical University Hospital, Taipei, Taiwan; 2 Division of Gastroenterology, Department of Internal Medicine, General Cathay Hospital, Taipei, Taiwan; 3 Department of Public Finance, National Taipei University, Taipei, Taiwan; 4 School of Health Care Administration, Taipei Medical University, Taipei, Taiwan; 5 Division of Urology, Department of Surgery, Far Eastern Memorial Hospital, New Taipei City, Taiwan; Roswell Park Cancer Institute, United States of America

## Abstract

**Background:**

This study attempted to reveal the incidence and risk of synchronous and metachronous esophageal cancer in subjects with oral, oropharyngeal and hypopharyngeal cancer based on a population-wide database in Taiwan.

**Methods:**

We retrieved data for this cross-sectional study from the Taiwanese Longitudinal Health Insurance Database 2000. The study group included 2,965 subjects who had received their first-time diagnosis of oral/oropharyngeal/hypopharyngeal cancer in 2002∼2009. We assigned the date of their first diagnosis of oral/oropharyngeal/hypopharyngeal cancer as the index date. We also randomly retrieved 29,650 comparison subjects matched with the study subjects in terms of gender and age group. We assigned their first medical utilization that occurred in the index year as the index date for the comparison group. We further performed a conditional logistic regression to investigate the association between esophageal cancer and oral cancer.

**Results:**

Results showed that prevalences of esophageal cancer within 3 months before and after the index date were respectively 2.19% and 0.04% for the study and comparison groups. A conditional logistic regression revealed that the odds ratio (OR) of esophageal cancer for subjects with oral/oropharyngeal/hypopharyngeal cancer was 55.33 (95% confidence interval (CI): 29.86∼102.52) compared to comparison subjects. Furthermore, compared to comparison subjects, ORs for esophageal cancer were respectively 18.41 (95% CI: 8.50–39.85), 40.49 (95% CI: 15.11∼108.64), and 240.96 (95% CI: 125.49–462.69) for study subjects with a malignancy of the oral cavity, oropharynx, and hypopharynx.

**Conclusion:**

We concluded that there were relatively high chances for synchronous and metachronous esophageal cancers being detected through panendoscopy in patients with oral, oropharyngeal, and hypopharyngeal cancers. The routine use of panendoscopy in such patients should be encouraged with a higher priority.

## Introduction

Oral, oropharyngeal and hypopharyngeal cancers are major health threats in many Asian countries. With the popularized use of betel nuts, along with smoking and alcohol consumption, incidences of oral, oropharyngeal and hypopharyngeal cancers in these countries far exceed global averages. In Taiwan, according to the latest government cancer registry data, the annual incidence of oral, oropharyngeal and hypopharyngeal cancers reached 40.8 per 104 people in 2009 [1]. These cancers in most cases share a common carcinogenetic pathway of surface mucosal exposure to carcinogens originating from betel nut, cigarettes, and alcohol. This led to the notorious phenomenon called “field cancerization” of the upper aerodigestive tract [2]–[5]. Carcinogens tend to disperse along the digestive tract and result in multifocal cancerous lesions. However, huge diversity exists between the reported incidences for synchronous and metachronous esophageal cancers. With such inconclusive results, some researchers have questioned the efficacy and cost-benefit of the routine use of panendoscopy to detect synchronous and metachronous esophageal cancers [6]–[9].

This study attempted to reveal the incidence and risk of synchronous and metachronous esophageal cancer in patients with oral, oropharyngeal and hypopharyngeal cancers based on a population-wide database. Results from our study can be regarded as a guideline reference for the diagnosis and management of patients with head and neck cancers.

## Methods

### Database

We retrieved data for this cross-sectional study from the Longitudinal Health Insurance Database (LHID2000). Taiwan began its National Health Insurance (NHI) program in 1995. The NHI program is characterized by universal coverage, a single-payer payment system with the government as the sole insurer, comprehensive benefits, access to any medical institution of the patient’s choice, and a wide variety of providers well distributed throughout the country. The enrollment rate has remained about 97% of the Taiwanese population since its initiation. The LHID2000 includes original medical claims data and registry files for 10^6^ individuals randomly sampled from all enrollees in the NHI program in 2000 (approximately 23.72 million enrollees). Some researchers and the Taiwan National Health Research Institute have demonstrated the high validity of the data from the NHI program [10], [11]. Furthermore, plenty of studies employing data from the Taiwan NHI have been published in internationally peer-reviewed journals [12], [13].

As the LHID2000 consists of de-identified secondary data released to the public for research purposes, after consulting with the director of the Institutional Review Board (IRB), this study was exempted from full review and approved by the Taipei Medical University IRB.

### Study Population

This cross-sectional study included study and comparison groups to compare prevalences of esophageal cancer between these two groups. We selected the study group by identifying 3011 subjects who had visited outpatient care centers or hospitalized and had received their first-time diagnosis of oral, oropharyngeal or hypopharyngeal cancers (ICD-9-CM codes 141, 143, 144. 145, 146, 148 or 149) between January 1, 2002 and December 31, 2009. We excluded those subjects aged less than 18 years (*n* = 26) in order to include only the adult population. We further assured that all included subjects had received an oral/oropharyngeal/hypopharyngeal cancer diagnosis following an biopsy in order to better ensure the validity of the oral cancer diagnosis. As a result, 2965 subjects with oral, oropharyngeal or hypopharyngeal cancers were included in the study group. We assigned the date of their first diagnosis of oral cancer as the index date.

We likewise selected a comparison group from the remaining enrollees of the LHID2000. A ratio of 1∶10 was used to randomly retrieve 29,650 comparison subjects matched with the study subjects in terms of gender, age group (18∼29, 30∼39, 40∼49, 50∼59, 60∼69, and >69 years), and year of the index date. We chose ten comparison subjects for each study subject on account of the very low prevalence of esophageal cancer. Therefore, this large sample size allowed us to detect a real difference in the prevalence of esophageal cancer between the study and comparison groups. In the study group, the year of the index date was the year in which the study subjects received their first diagnosis of oral, oropharyngeal or hypopharyngeal cancers, while for the comparison group, the year of index date was simply a matched year in which comparison subjects had visited a doctor. We assigned the date of their first doctor visits occurring during the matched year as the index date for the comparison group.

### Exposure Assessment

We identified esophageal cancer cases based on ICD-9-CM codes 150 or 150.0∼150.9. Additionally, since the implication of the study is to tell whether the routine use of panendoscopy in patients with oral, oropharyngeal and hypopharyngeal cancers should be encouraged, we only included esophageal cancer cases if they had received an esophageal cancer diagnosis following an endoscopic biopsy. In this study, we compared the prevalence of esophageal cancer within 3 months before and after the index date between the study and comparison groups.

### Statistical Analysis

This study used the SAS system (SAS System for Windows, vers. 8.2, SAS Institute, Cary, NC) to analyze the data. A χ^ 2^ test was performed to compare prevalences of esophageal cancer within 3 months before and after the index date between the study and comparison groups. We further performed a conditional logistic regression (conditioned on age group, gender, and index year) to investigate the association between esophageal cancer and oral/oropharynx/hypopharynx cancer. We used the conventional *p* ≤ 0.05 as statistical significance in this study.

## Results

Of the 32,615 sampled subjects, the mean age was 55.0 (±13.9) years, and only 11.3% were <40 years old. In addition, the overwhelming majority (83.9%) were males. Of the study subjects, 1893 (63.8%), 372 (12.6%), 450 (15.2%), and 250 (8.4%) had a malignancy located in the oral cavity, oropharynx, hypopharynx, and unspecified, respectively.

Table 1 presents prevalences of esophageal cancer within 3 months before and after the index date between the study and comparison groups. After matching for age group and gender, prevalence of esophageal cancer for all sampled subjects was 0.24%, while they were 2.19% and 0.04% for the study and comparison groups, respectively. The χ^ 2^ test showed that there was a significant difference in the prevalences of esophageal cancer between the study and comparison groups (*p*<0.001). Table 1 also displays the odds ratios (ORs) of esophageal cancer between these two groups. A conditional logistic regression (conditioned on age group, gender, and the index year) showed that the OR of esophageal cancer for subjects with oral, oropharyngeal and hypopharyngeal cancers was 55.33 (95% confidence interval (CI): 29.86∼102.52, *p*<0.001) compared to comparison subjects.

**Table 1:** Prevalence rates and odds ratios for esophageal cancer among sample subjects with oral cancer and comparison subjects.

Presence of esophageal cancer	Total sample *N* = 32,615		Subjects with oral cancer *N* = 2965		Comparison subjects *N* = 29,650	
	No.	Percent (%)	No.	Percent (%)	No.	Percent (%)
Yes	77	0.24	65	2.19	12	0.04
Odds ratio^ a^ (95% confidence interval)	–		55.33*** (29.86∼102.52)		1.00	

Notes: ^a^The odds ratio was calculated using the conditional logistic regression model (conditioned on age group and gender);

*** *p*<0.001.

Table 2 further presents the ORs of esophageal cancer between these two groups by cancer location. Since 5 study subjects with esophageal cancer simultaneously had 2 or 3 locations of oral cancer, we did not include these 5 subjects in the analysis in Table 2. A conditional logistic regression (conditioned on age group, gender, and the index year) showed that compared to comparison subjects, the ORs for esophageal cancer were 18.41 (95% CI: 8.50∼39.85), 40.49 (95% CI: 15.11∼108.64), and 240.96 (95% CI: 125.49∼462.69) for study subjects with a malignancy of the oral cavity, oropharynx, and hypopharynx, respectively.

**Table 2:** Prevalence rates and odds ratios for esophageal cancer among sample subjects by oral cancer location.

Presence of esophageal cancer	Subjects with oral cancer						Comparison subjects *N* = 29,650	
	Oral cavity *N* = 1893		Oropharynx *N* = 372		Hypopharyngeal *N* = 450			
	No.	Percent (%)	No.	Percent (%)	No.	Percent (%)	No.	Percent (%)
Yes	14	0.74	6	1.61	40	8.89	12	0.04
Odds ratio^ a^ (95% confidence interval)	18.41*** (8.50∼39.85)		40.49*** (15.11∼108.64)		240.96*** (125.49∼462.69)		1.00	

Notes: ^a^Odds ratios were calculated using the conditional logistic regression model (conditioned on age group and gender);

*** *p*<0.001.

## Discussion

Results of our study revealed that there is strong evidence supporting esophageal cancer tending to occur simultaneously or at least metachronously in patients with oral, oropharyngeal and hypopharyngeal cancers. This is the first time that a nationwide, universal coverage database was used. Considering the high prevalence of the betel nut-chewing habit in Taiwan [14], our study should be able to provide very strong evidence for clarifying the relationship between these carcinogen exposure-related cancers.

Since the issue of synchronous and metachronous esophageal cancer in patients with oral, oropharyngeal and hypopharyngeal cancers was first raised by Slaughter et al. in 1953, many researchers have attempted to explore the relationship between these cancers [15]–[17]. Traditionally, the endoscopic procedure was a reasonable choice to detect synchronous and metachronous carcinomas [18]–[20]. Many researchers used a panendoscopic examination in the routine evaluation of newly diagnosed head and neck cancer patients. Shiozaki et al. reported a 5.1% incidence of synchronous or metachronous asymptomatic esophageal cancer among patients with head and neck cancers [21]. Tincani et al. reported the rate to be 8.3% [22]. In a recent report by Lee et al. in 2010, 30.4% of patients with documented head and neck cancers were confirmed to have esophageal neoplasia [23]. Incidences significantly varied possibly due to the limited number of enrolled cases. So far, the largest enrolled number of similar study was 3375 patients reported by Shibuya et al. with a resultant incidence of 2.4% [24].

Results from most of the above studies revealed that the incidence of a synchronous or metachronous esophageal cancer was significant higher when the initial diagnosed cancer was located in the hypopharynx, compared to the oral cavity, oropharynx, or larynx. Since endoscopy appears to be a much more-sensitive tool for detecting esophageal cancer, some doctors recommend the routine use of esophagoscopy for those patients with a squamous cell carcinoma of the hypopharynx [6]. However, a major problem in the previous studies was that most of them were case-control studies, and the patients were slated to receive esophagoscopy after the initial diagnosis of oral and pharyngeal cancers. The experimental design and settings raise concerns of a significant surveillance bias. In reality, this surveillance bias is difficult to eliminate due to the fact that it is very unlikely one could initiate a large prospective cohort with individuals in the control group who have received routine panendoscopy, even when environmental exposures are matched. Various factors are related to oral and esophageal cancers, and incorporating all of these factors into the analysis is needed in studies which aim to justify the “field cancerization” theory or justify influences of a factor “contributing to oral/oropharyngeal/hypopharyngeal cancer” to “getting synchronous/metachronous esophageal cancer”, and therefore instead of researching the increase in incidence of getting synchronous/metachronous esophageal cancer, we analyzed our nationwide database to see if quite a number of synchronous and metachronous esophageal cancers were diagnosed through panendoscopy compared to a gender- and age-matched baseline population.

Many epidemiological studies have attributed drinking and smoking to the phenomenon of field cancerization for more than five decades [3], [25]–[29]. In brief, field cancerization of the mucous membranes of the aerodigestive tract frequently develops in response to tobacco and alcohol usage. This phenomenon is characterized by a variety of premalignant and malignant epithelial changes that may lead to the development of multiple primary cancers of the aerodigestive tract. Results from all of these related studies imply that cancers from the upper aerodigestive tract less often occur solitarily, and especially when topical carcinogen exposure is to blame, lesions tend to arise from multiple sites with similar carcinogenesis environments. These associations appear to be logically reasonable. As a substances passes along the upper aerodigestive tract either through ingestion or inhalation, mucosal surfaces inevitably are exposed to the carcinogens. Muto et al. identified that the interaction between drinking and a deficiency of the alcohol dehydrogenase type 2 (ALDH2) allele increases the risk of esophageal cancer [30]. A deficiency of the enzyme, aldehyde dehydrogenase, especially seen in patients of East Asian descent, results in the reduced metabolism of acetaldehyde which is known to have cancer-promoting effects [31]. This phenomenon was further confirmed by Brooks et al. [32] however, those authors stated that an ALDH2 deficiency does not influence esophageal cancer risk in non-drinkers, implying that topical carcinogen exposure is essential in the pathogenesis of these patients. There is already evidence showing that deficiencies in ALDH2 are associated with an increased risk of oral cancers [33], [34]. In normal conditions, it is nearly impossible to have alcohol exposure to only one sub-site in the upper aerodigestive tract. According to findings from epidemiological studies to genetic studies, it is therefore crucial for physicians practicing in these fields to understand the true relationships between different cancers that arise from the upper aerodigestive tract.

As mentioned before, the most straightforward strategy to detect esophageal cancer is through panendoscopy. Recently, a study by Wang et al. focused on evaluating the feasibility and safety of unsedated transnasal endoscopy (TNE) for screening patients with head and neck squamous cell carcinoma, who are also at high risk for synchronous and/or metachronous esophageal cancers [35]. They found that esophageal squamous cell carcinomas and high-grade intraepithelial neoplasms were detected in 10.1% and 7.3%, respectively, of cases receiving TNE screening. More importantly, the completion rate of TNE in head and neck cancer was as high as 96.7%. The result is highly suggestive that unsedated TNE is safe and feasible for screening synchronous or metachronous esophageal neoplasms in high-risk patients. This important finding might lead us to reevaluate the rationale of using panendoscopy as a screening tool, which was previously regarded as not being cost-effective and less compliable for patients.

Some limitations of our study should be addressed. First, our study used the data based on the International Classification of Diseases Ninth Revision (ICD-9). Through the ICD-9 codes, while the existence of esophageal cancer can be confirmed, it is difficult to tell if the cancer was truly a primary cancer or the cancer originated from local spread or even metastasis. Esophageal cancers rarely metastasize to oral-maxillary regions and vice versa; secondary esophageal metastasis from the oral cavity and pharynx is also very rare [36]–[38]. However, we were unable to exclude rare cases in which synchronous or metachronous cancer was in fact from a direct extension or metastasis of a primary cancer, and this resulted in a certain bias in interpreting the retrieved data. The best we can do is to limit the diagnosis of the esophageal cancer to those “following an endoscopic biopsy”. We believe that with this limitation, esophageal cancers resulting from obvious direct extensions of hypopharyngeal cancer would be largely ruled out as in such cases, a diagnosis of esophageal cancer would more likely be made at the time of the hypopharynx biopsy through a rigid laryngoscope/esophagoscope and less likely to be made after flexible panendoscopy.

Second, the ICD-9-CM codes for oral, pharyngeal, and esophageal cancers are based on tumor sites and do not reveal the histological types of the cancer. The database in this study might have included patients with oral, pharyngeal, and esophageal cancers of, for example, an adenocarcinoma, which was proven to less likely be related to exposure to betel-quid carcinogens [39]. Nevertheless, we believe that due to the fact that nearly 90% of the oral, oropharyngeal and hypopharyngeal cancers and over 90% of esophageal cancers are the squamous cell carcinoma type in Taiwan, the conclusions based on data interpretation in our study remain unchanged. However the lack of information on histology is obviously a limitation of our study and prevents any further analysis of this issue.

Third, we were unable to have a comparison control group that consisted of patients who had a documented oral examination and endoscopy with no evidence of malignancy at the baseline. It is clear that the above-mentioned examinations were not applied at the baseline to the 29,650 comparison subjects. Therefore, it is possible that this may have caused our study to suffer from an ascertainment bias, in which the association detected in this study could be partly explained by subjects with oral cancer being more likely to be diagnosed with esophageal cancer purely on account of being sicker and their subsequent increased exposure to the medical community.

Lastly, we cannot rule out the possibility of including comparison subjects who had oral cancer on the index date because comparison subjects were not given a thorough oral examination. Therefore, it is possible that some of the comparison subjects may also have been suffering from oral cancer. However, if such a bias exists in the data, the results of our analysis would be biased towards the null.

Our study results suggested that relatively high chance of synchronous and metachronous esophageal cancers to be detected through panendoscopy in patients with oral, oropharyngeal and hypopharyngeal cancers. According to the findings revealed in our study, we know that these types of cancers tend to occur together or at least within a relatively short period of time. We suggest that the routine use of panendoscopy in patients with hypopharyngeal cancer be encouraged with a higher priority. Even further, if possible, it should be applied to all patients with cancers arising from the upper aerodigestive tract.
