# Correction: Routine Endoscopy for Esophageal Cancer Is Suggestive for Patients with Oral, Oropharyngeal and Hypopharyngeal Cancer

**DOI:** 10.1371/journal.pone.0170866

**Published:** 2017-01-20

**Authors:** Shih-Han Hung, Ming-Chieh Tsai, Tsai-Ching Liu, Herng-Ching Lin, Shiu-Dong Chung

The affiliation for the first author is incorrect. Shih-Han Hung is affiliated with: Department of Otolaryngology, Taipei Medical University Hospital, Taipei, Taiwan; Graduate Institute of Medical Sciences, College of Medicine, Taipei Medical University, Taipei, Taiwan; and Department of Otolaryngology, School of Medicine, College of Medicine, Taipei Medical University, Taipei, Taiwan.
